# Supplementary material for: Contextualising the developability risk of antibodies with lambda light chains using enhanced therapeutic antibody profiling
Source: Commun Biol. 2024 Jan 8;7:62. doi: 10.1038/s42003-023-05744-8 (PMC10774428; doi:10.1038/s42003-023-05744-8)
Supplement: Supplementary file 2 — Supplementary Information [file 42003_2023_5744_MOESM2_ESM.pdf]

Supplementary Information

Contextualising the developability risk of antibodies with lambda light chains using enhanced therapeutic antibody profiling

Matthew I. J. Raybould, Oliver M. Turnbull, Annabel Suter, Bora Guloglu, Charlotte M. Deane*

Oxford Protein Informatics Group, Department of Statistics, University of Oxford, 24-29 St Giles', Oxford, OX1 3LB, United Kingdom.

*Corresponding author. E-mail: deane@stats.ox.ac.uk

Supplementary Notes

Supplementary Note 1: Evaluating ABodyBuilder2's Performance on Unseen Therapeutics

To ensure that ABodyBuilder2's improved general performance translates to clinical-stage therapeutics (CSTs), we mined Thera-SAbDab to identify eight CST variable regions (Fvs) whose first sequence identical X-ray crystal structures were released after 31st July 2021 (Supplementary Table 1); the 119 CST Fvs with crystal structures released before this date (Table S2) would not provide a fair indication of expected performance as they would have formed part of the ABodyBuilder2 training set.

Evaluating the performance of ABodyBuilder2 and ABodyBuilder1 over these eight 'unseen' CSTs we found that ABodyBuilder2's accuracy (μ_{CDRH3}: 2.68 Å) was markedly better than ABodyBuilder1's (μ_{CDRH3}: 3.32 Å; see Supplementary Table 1 for all CDRs). We also observed that ABodyBuilder2's higher backbone prediction accuracy across this subset of CSTs translated into an improved accuracy in the proportions of side chains assigned as buried/exposed, a key parameter in evaluating the structure-dependent TAP metric values (ABodyBuilder2: 96.39% accuracy, ABodyBuilder1: 95.99% accuracy). Improvement was further magnified when considering

only the formal IMGT CDR [1] residues. Together with the results from the ImmuneBuilder publication [2], this evidence motivated a change in the TAP protocol to use ABodyBuilder2 for structural modelling in place of ABodyBuilder1.

Supplementary Figures and Tables

The following pages contain **16** Supplementary Figures and **10** Supplementary Tables.

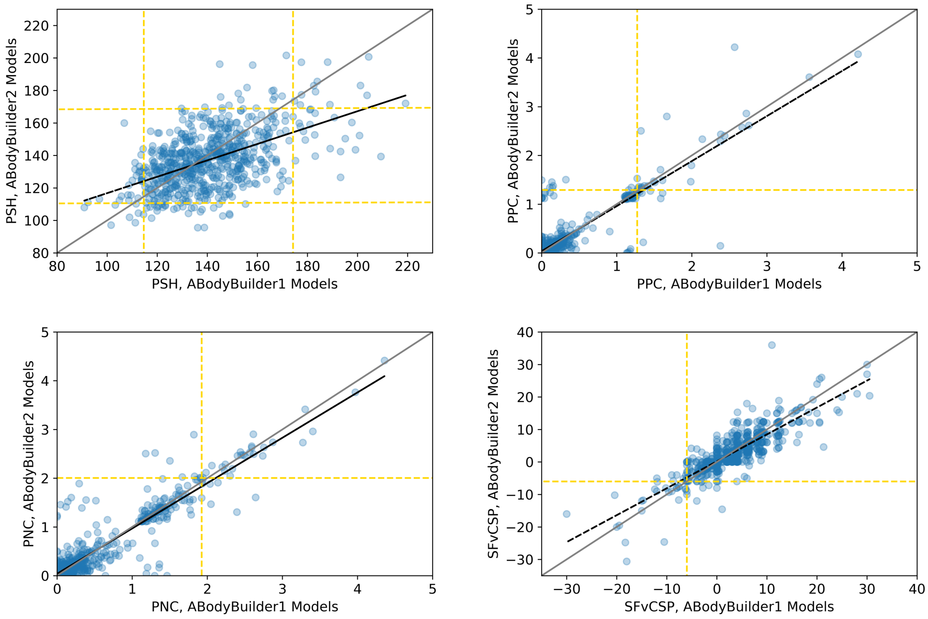

Supplementary Fig. 1 Scatterplots showing the degree of consistency in TAP metric values and thresholds over all CSTs when evaluated on an ABodyBuilder1 (x-axis) or ABodyBuilder2 (y-axis) model. Amber thresholds based on the 5th and/or 95th percentile values for each modelling tool are shown with dashed lines. A least-squares line of best fit for each metric is shown in black, with x=y in grey.

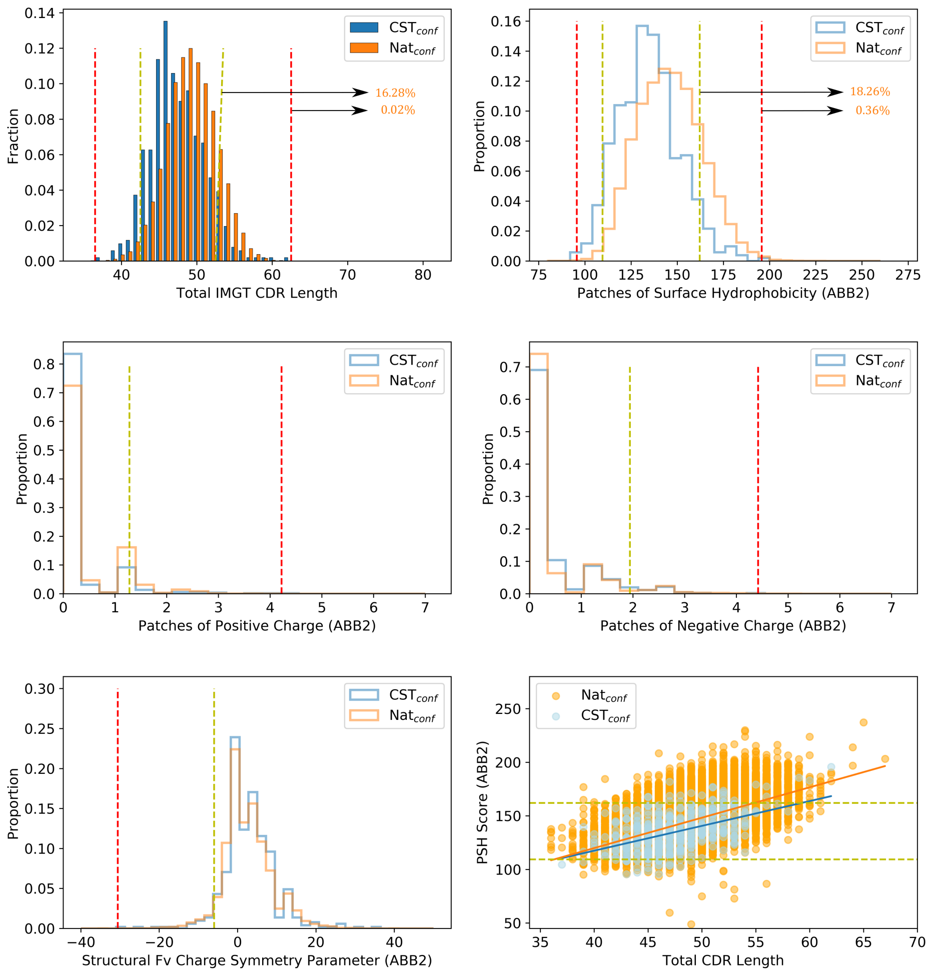

Supplementary Fig. 2 The five TAP developability metrics calculated over the set of CDRH3 confidence-filtered CSTs (CST_{conf}, blue) and natural human antibodies (Nat_{conf}, orange). Amber and red flagging thresholds are calculated based on the CST_{conf} subset. The percentages of Nat_{conf} antibodies surpassing the upper Total CDR Length and Patches of Surface Hydrophobicity thresholds are highlighted.

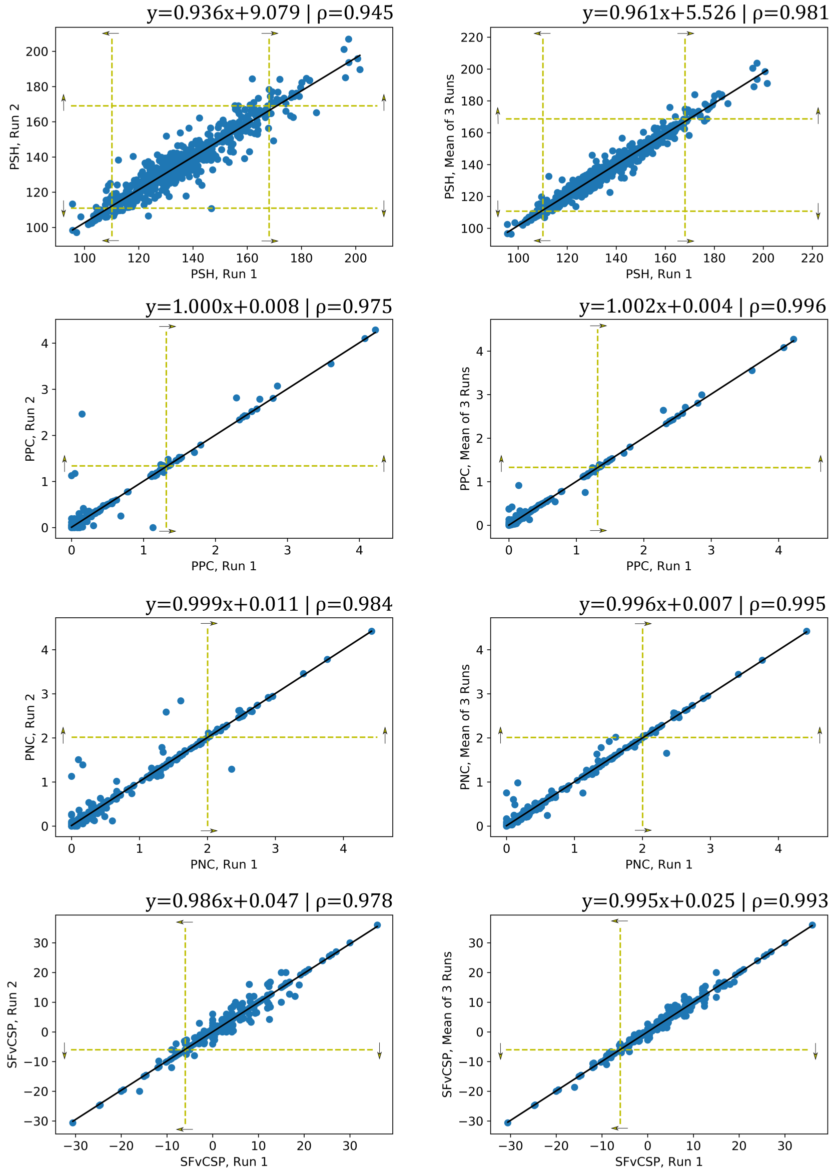

Supplementary Fig. 3 The variation in TAP scores per CST when comparing (left column) one independent ABodyBuilder2 prediction to another, and (right column) one independent ABodyBuilder2 prediction to the mean of three predictions. Amber thresholds are calculated based on the 5th and/or 95th percentile values for each run/set of runs. Arrows indicate the flagging region relative to each threshold. Least-squares regression lines are plotted with the corresponding equation displayed above each figure.

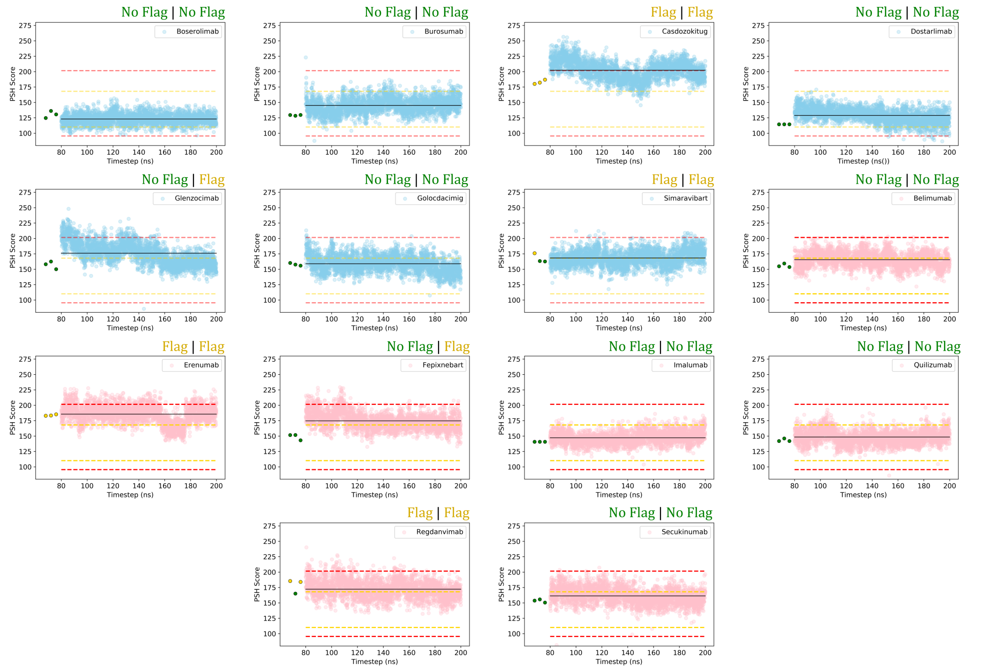

Supplementary Fig. 4 Analysing the Patches of Surface Hydrophobicity (PSH) score for 14 CSTs calculated every 0.04ns over the final 120ns of a 200ns molecular dynamics simulation. The blue trajectories show CSTs that did not have solved Fv structures in the ABodyBuilder2 training set, while pink trajectories show CSTs that did. The amber and red flagging thresholds from Table 1 are shown as dashed lines in corresponding colours; the mean PSH value across the simulation is represented by a solid black line. The PSH values obtained by running TAP on three independent ABodyBuilder2 predictions are shown as spots before the simulation, coloured by the assigned flag. The annotations at the top-right of each graph are in the format [Flag assigned based on the ensemble of three TAP calculations] — [Flag assigned based on the simulation mean value].

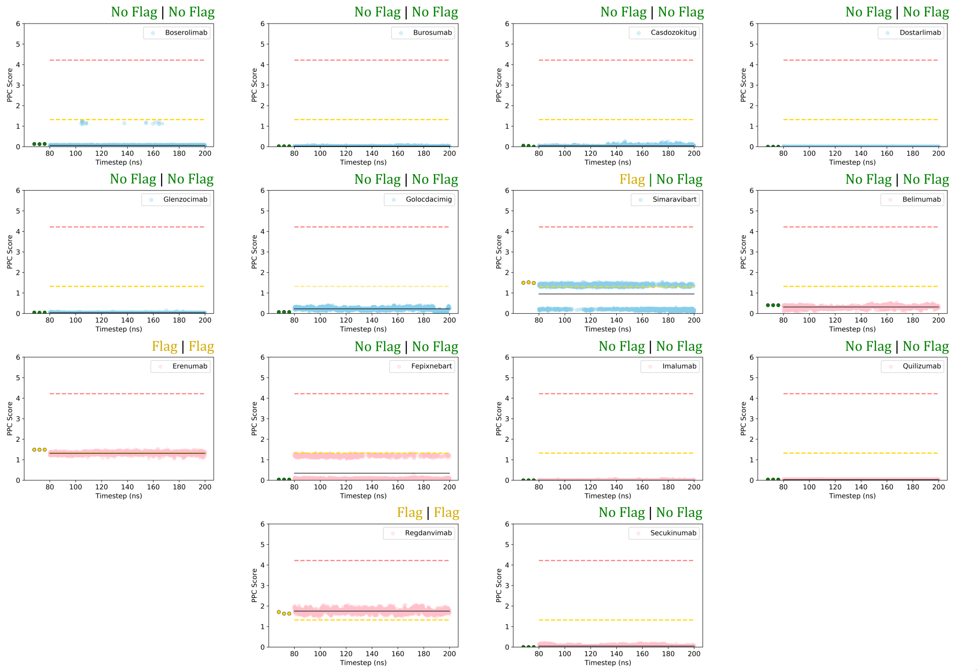

Supplementary Fig. 5 Analysing the Patches of Positive Charge (PPC) score for 14 CSTs calculated every 0.04ns over the final 120ns of a 200ns molecular dynamics simulation. The blue trajectories show CSTs that did not have solved Fv structures in the ABodyBuilder2 training set, while pink trajectories show CSTs that did. The amber and red flagging thresholds from Table 1 are shown as dashed lines in corresponding colours; the mean PSH value across the simulation is represented by a solid black line. The PSH values obtained by running TAP on three independent ABodyBuilder2 predictions are shown as spots before the simulation, coloured by the assigned flag. The annotations at the top-right of each graph are in the format [Flag assigned based on the ensemble of three TAP calculations] — [Flag assigned based on the simulation mean value].

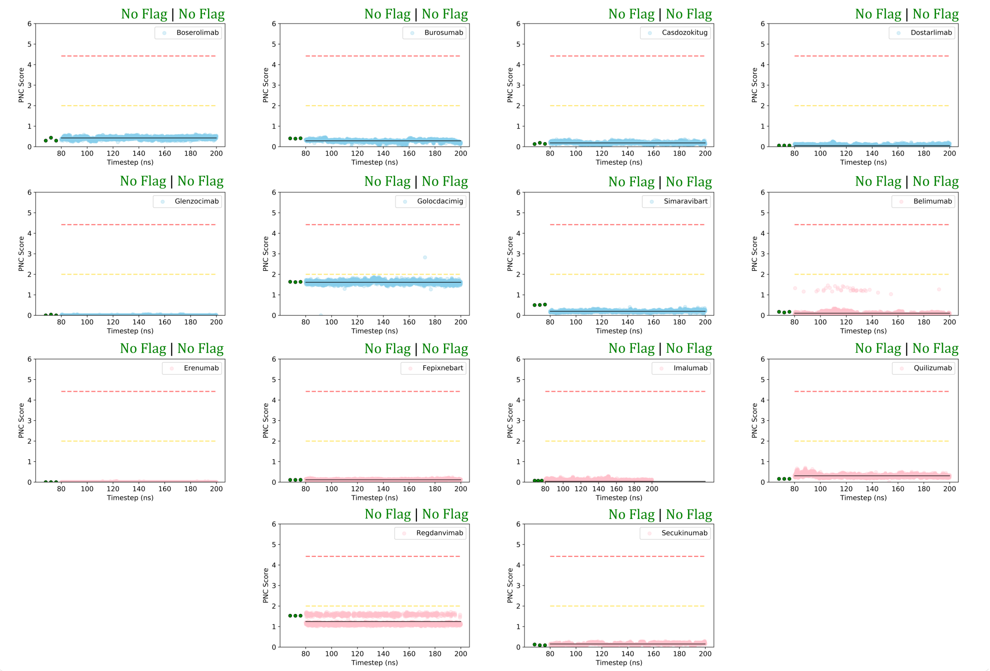

Supplementary Fig. 6 Analysing the Patches of Negative Charge (PNC) score for 14 CSTs calculated every 0.04ns over the final 120ns of a 200ns molecular dynamics simulation. The blue trajectories show CSTs that did not have solved Fv structures in the ABodyBuilder2 training set, while pink trajectories show CSTs that did. The amber and red flagging thresholds from Table 1 are shown as dashed lines in corresponding colours; the mean PSH value across the simulation is represented by a solid black line. The PSH values obtained by running TAP on three independent ABodyBuilder2 predictions are shown as spots before the simulation, coloured by the assigned flag. The annotations at the top-right of each graph are in the format [Flag assigned based on the ensemble of three TAP calculations] — [Flag assigned based on the simulation mean value].

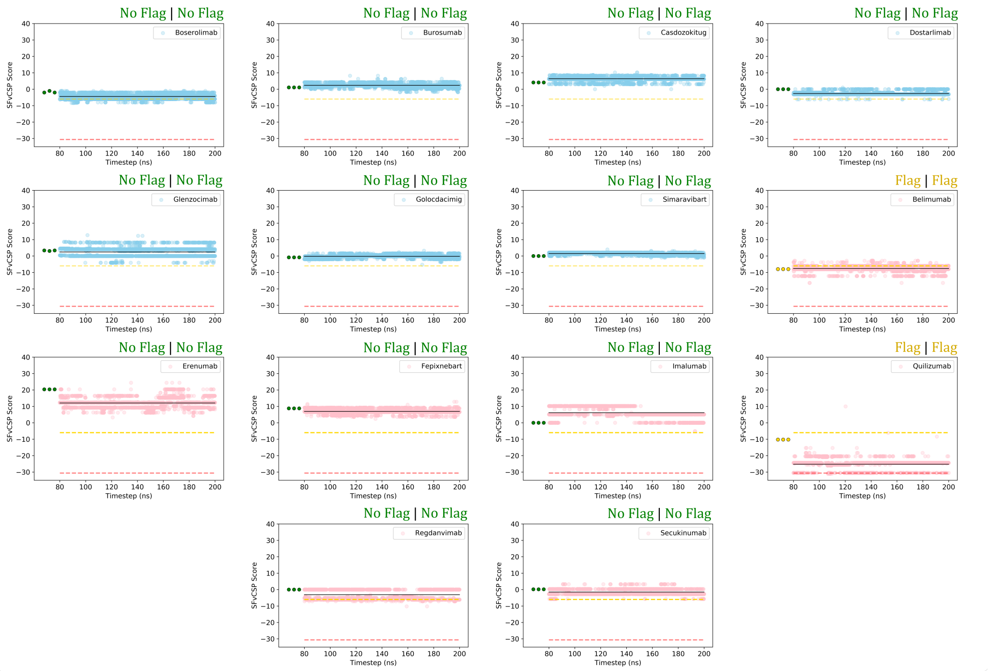

Supplementary Fig. 7 CSTs calculated every 0.04ns over the final 120ns of a 200ns molecular dynamics simulation. The blue trajectories show CSTs that did not have solved Fv structures in the ABodyBuilder2 training set, while pink trajectories show CSTs that did. The amber and red flagging thresholds from Table 1 are shown as dashed lines in corresponding colours; the mean PSH value across the simulation is represented by a solid black line. The PSH values obtained by running TAP on three independent ABodyBuilder2 predictions are shown as spots before the simulation, coloured by the assigned flag. The annotations at the top-right of each graph are in the format [Flag assigned based on the ensemble of three TAP calculations] — [Flag assigned based on the simulation mean value].

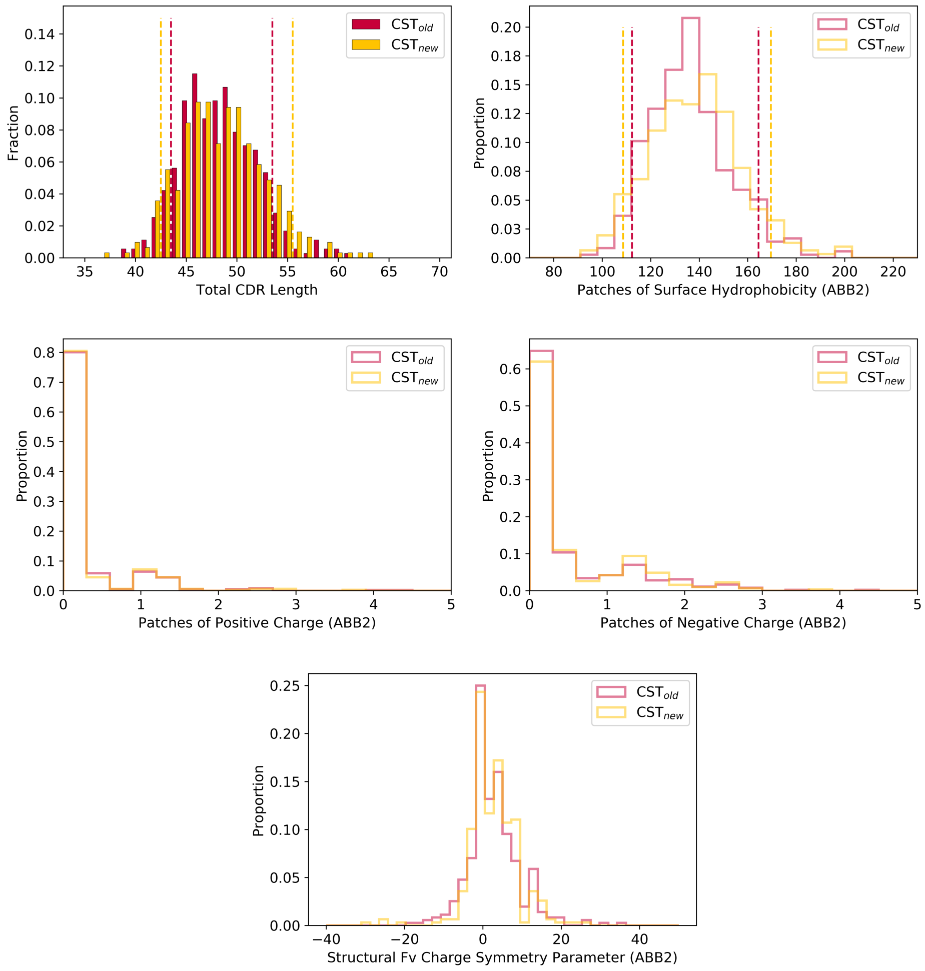

Supplementary Fig. 8 The five TAP developability metrics calculated over the set of CSTs recognised by the WHO between 1987-2017 (CST$_{\text{old}}$), and the set recognised between 2018-Present (CST$_{\text{new}}$). The amber thresholds for the Total CDR Length and Patches of Surface Hydrophobicity (PSH) properties of each set are highlighted with dashed lines.

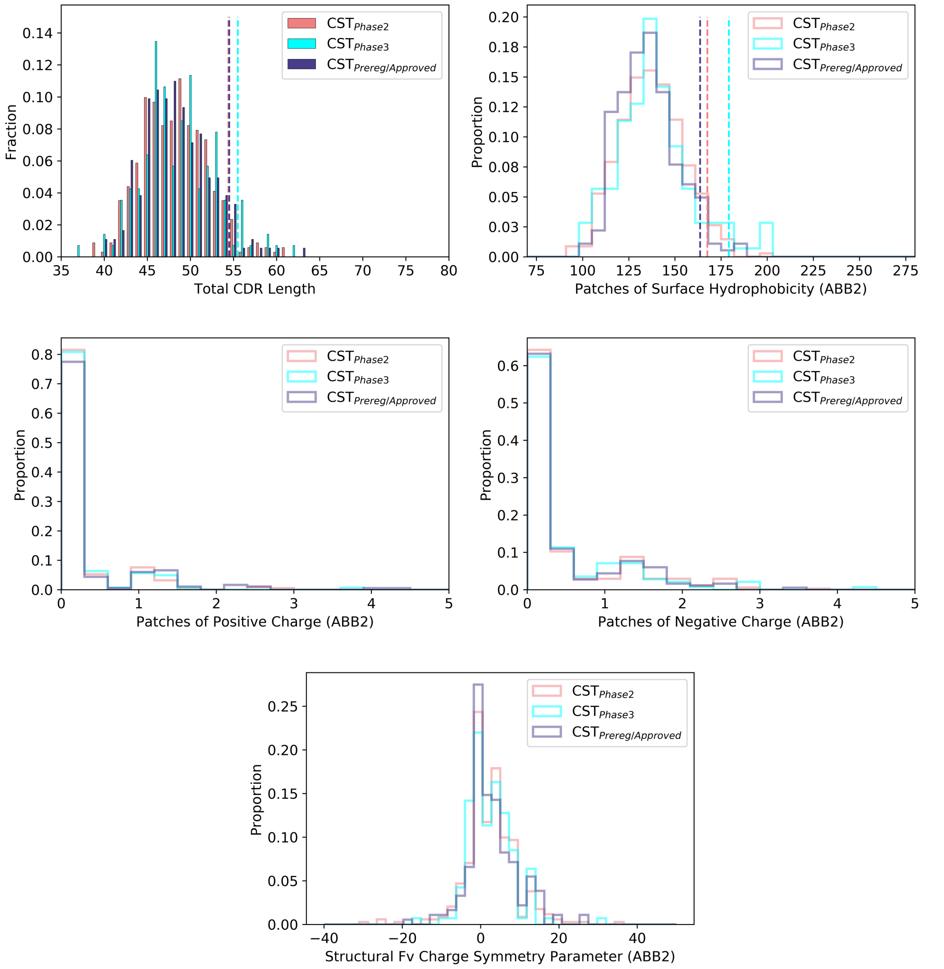

Supplementary Fig. 9 The five TAP developability metrics calculated over the set of CSTs in Phase-II clinical trials ($\mathrm{CST}_{\mathrm{Phase2}}$), the set in Phase-III clinical trials ($\mathrm{CST}_{\mathrm{Phase3}}$, and the set that have reached Preregistration/been approved as drugs ($\mathrm{CST}_{\mathrm{Prereg/Approved}}$). The amber thresholds for the Total CDR Length and Patches of Surface Hydrophobicity (PSH) properties of each set are highlighted with dashed lines.

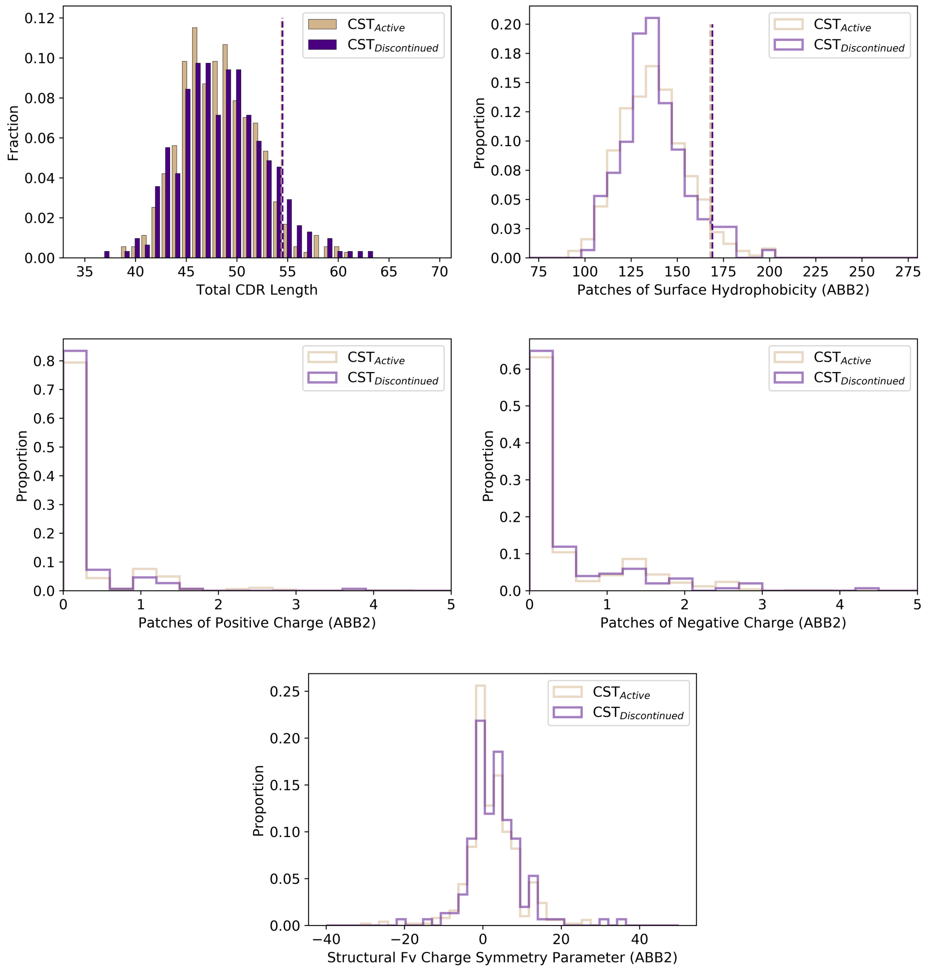

Supplementary Fig. 10 The five TAP developability metrics calculated over the set of CSTs in active development/that completed the development pipeline (CST_{Active}) and the set those development campaigns were terminated before approval ($CST_{Discontinued}$. The amber thresholds for the Total CDR Length and Patches of Surface Hydrophobicity (PSH) properties of each set are highlighted with dashed lines.

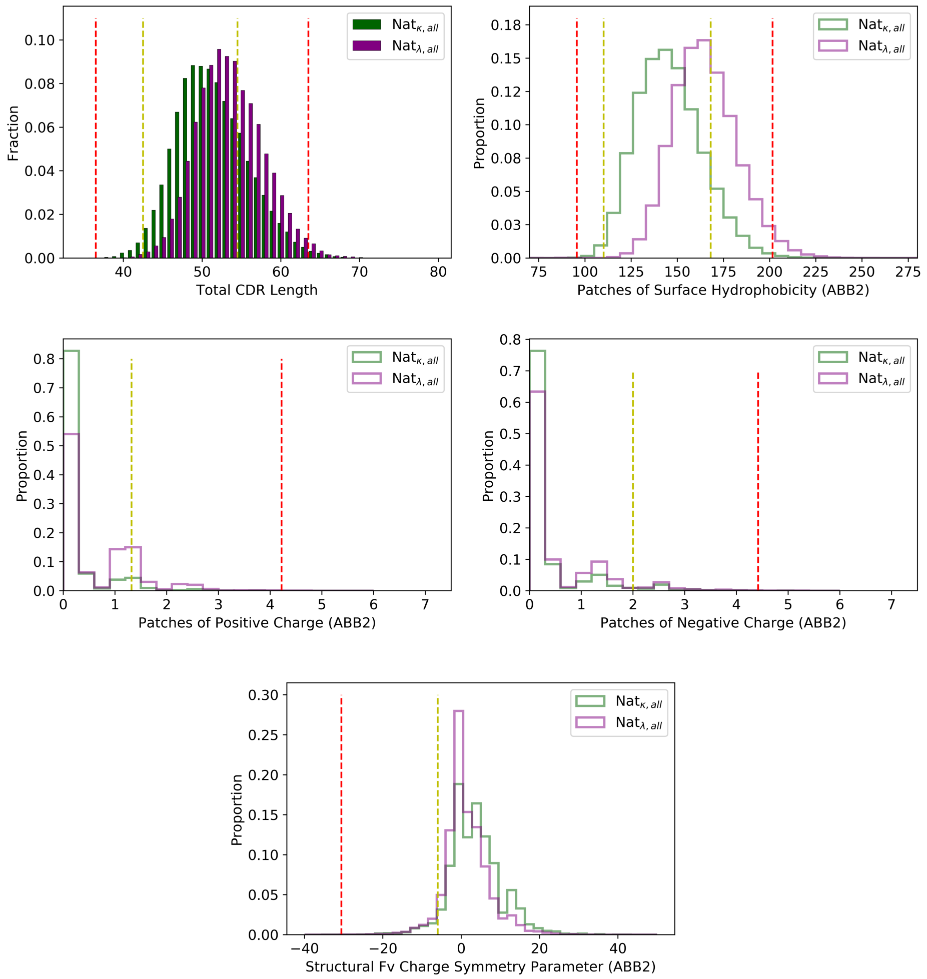

Supplementary Fig. 11 The five TAP developability metrics calculated over all natural human κ-antibodies and λ-antibodies.

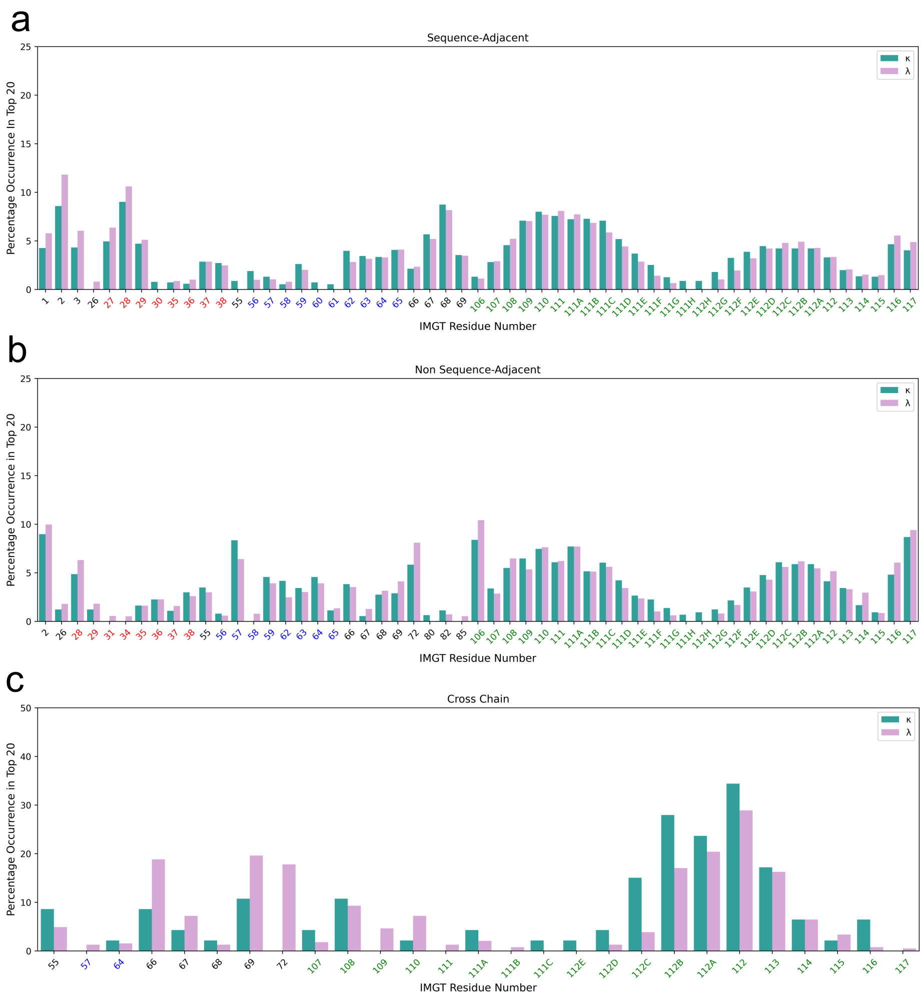

Supplementary Fig. 12 The percentage frequency that each heavy chain IMGT residue position occurs in the top-20-most hydrophobic (a) sequence adjacent and (b) sequence non-adjacent interactions amongst κ (seagreen) and λ (plum) red-flagging antibodies. (c) For each heavy chain position, the proportion of top-20-most hydrophobic sequence non-adjacent interactions involving that position that are cross-chain (i.e. involve a light chain residue). Residue numbers in the IMGT-defined CDR1 region are coloured red, in the IMGT-defined CDR2 regions are coloured blue, and in the IMGT-defined CDR3 regions are coloured green.

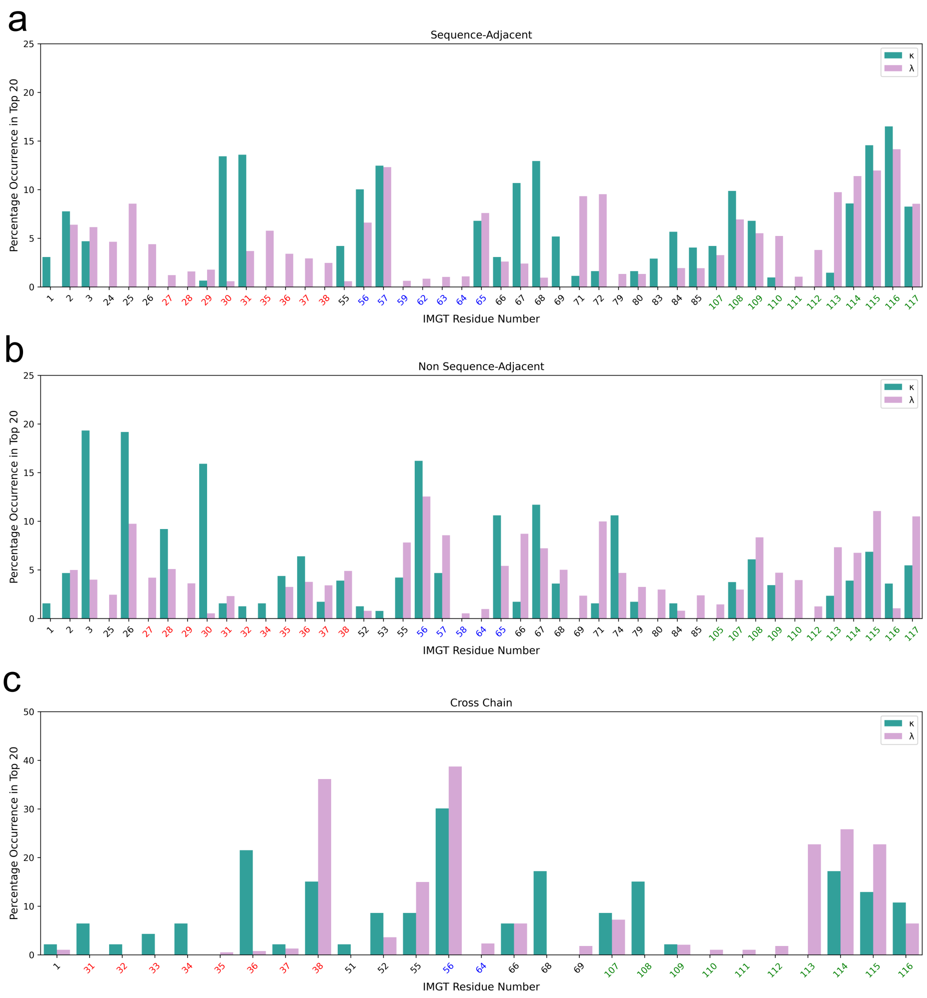

Supplementary Fig. 13 The percentage frequency that each light chain IMGT residue position occurs in the top-20-most hydrophobic (a) sequence adjacent and (b) sequence non-adjacent interactions amongst κ (seagreen) and λ (plum) red-flagging antibodies. (c) For each light chain position, the proportion of top-20-most hydrophobic sequence non-adjacent interactions involving that position that are cross-chain (i.e. involve a heavy chain residue). Residue numbers in the IMGT-defined CDR1 region are coloured red, in the IMGT-defined CDR2 regions are coloured blue, and in the IMGT-defined CDR3 regions are coloured green.

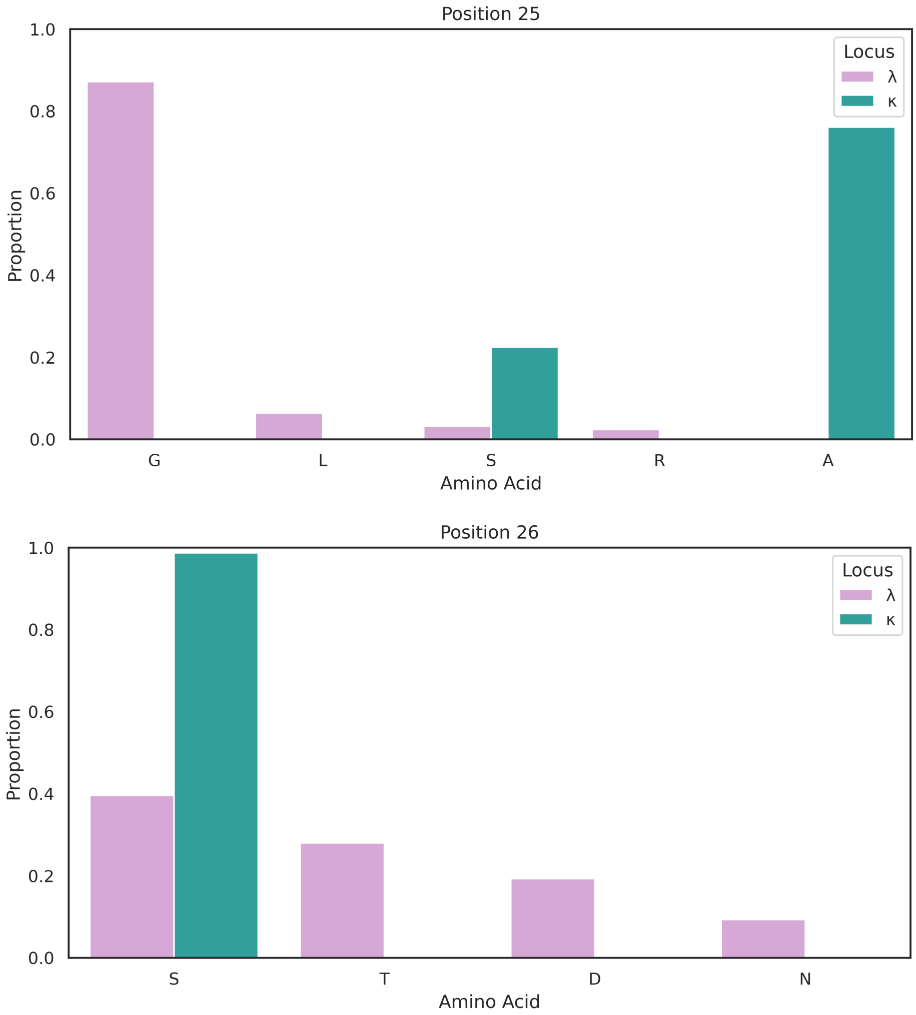

Supplementary Fig. 14 Bar charts showing the amino acid usages at IMGT positions 25 and 26 amongst natural λ-antibodies and natural κ-antibodies.

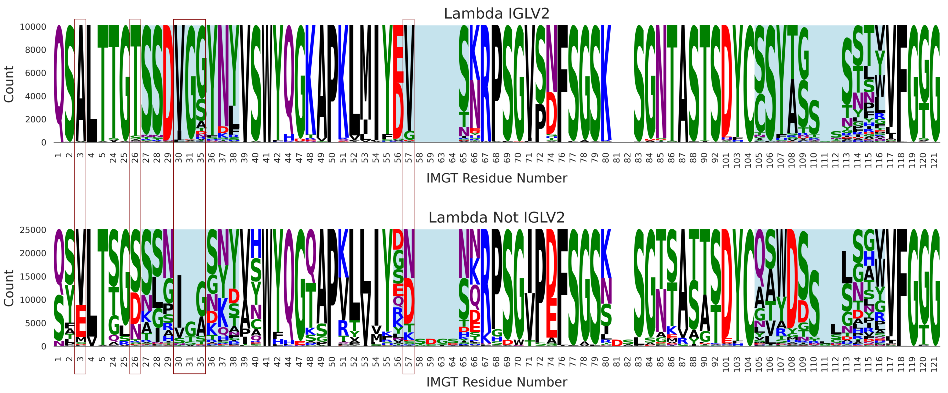

Supplementary Fig. 15 Sequence logo plots showing residue abundance across IGLV2 λ-antibodies and non-IGLV2 λ-antibodies by IMGT [1] residue position. Red-boxed positions are highlighted in the main manuscript.

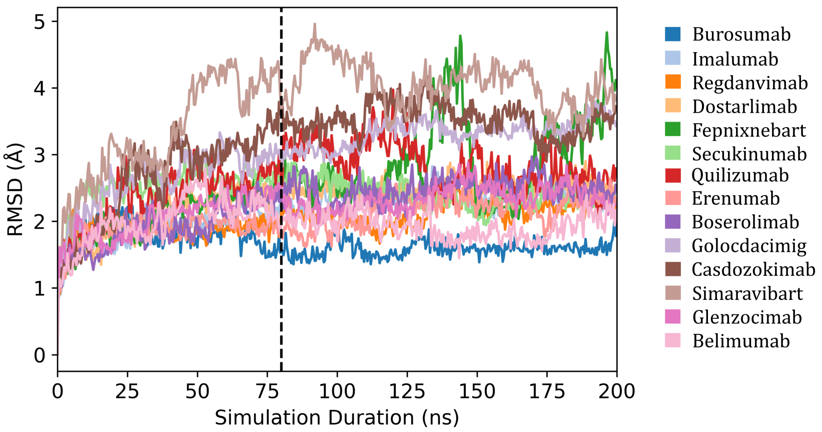

Supplementary Fig. 16 The root-mean squared deviation (RMSD) from the starting structure over the course of the 200 ns simulation for the 14 CSTs studied with molecular dynamics. TAP physicochemical properties were calculated on snapshots post-80 ns, once most simulations had begun to oscillate around a mean RMSD value.

**Therapeutic**	**PDB ID (Chains)**	**State, κ/λ, Resolution (Å)**
Boserolimab	8DS5 (CB)	Complex, κ, 1.93
Burosumab	7VEN (BA)	Apo, κ, 1.45
Casdozokitug	7ZXK (HL)	Complex, κ, 2.20
Cemiplimab	7WVM (AB)	Complex, κ, 3.40
Dostarlimab	7WSL (HL)	Complex, κ, 1.75
Glenzocimab	7R58 (HL)	Complex, κ, 1.90
Golocdacimig	7R8U (HL)	Complex, λ, 1.90
Simaravibart	7SBU (HL)	Complex, κ, 2.53

	Mean RMSD (Å)						% All S.E.R. Correct	% CDR S.E.R. Correct
	H1	H2	H3	L1	L2	L3		
ABB1	1.73	1.00	3.32	0.91	0.56	1.16	95.99%	94.14%
ABB2	0.78	0.95	2.68	0.77	0.50	0.80	96.39%	94.87%
% Improv.	*54.9%*	*5.0%*	*19.3%*	*5.4%*	*10.7%*	*31.0%*	*+0.40%*	*+0.73%*

Supplementary Table 1 (Above) The eight clinical-stage therapeutics (CSTs) with 100% sequence identity solved crystal structures publicly released after 31st July 2021 (ABodyBuilder2's training set cutoff date). (Below) The performance of ABodyBuilder1 [3] (ABB1) *vs.* ABodyBuilder2 [2] (ABB2) on this subset of CSTs. CDR: Complementarity-determining region; Improv: Improvement; S.E.R: Surface exposed residues.

Abituzumab (λ)	Adalimumab (κ)	Aducanumab (κ)	Alemtuzumab (κ)
Alomfilimab (κ)	Amivantamab (κ)	Amubarvimab (κ)	Andecalizumab (κ)
Anifrolumab (κ)	Arcitumumab (κ)	Atezolizumab (κ)	Avelumab (λ)
Bamlanivimab (κ)	Basiliximab (κ)	Bebtelovimab (λ)	Belimumab (λ)
Bentracimab (λ)	Benufutamab (κ)	Berlimatoxumab (κ)	Bevacizumab (κ)
Bezlotoxumab (κ)	Bimagrumab (λ)	Bococizumab (κ)	Briakinumab (λ)
Camrelizumab (κ)	Certolizumab (κ)	Cetuximab (κ)	Cinpanemab (λ)
Clesrovimab (κ)	Coltuximab (κ)	Conatumumab (κ)	Concizumab (κ)
Crenezumab (κ)	Crovalimab (κ)	Daclizumab (κ)	Daratumumab (κ)
Diridavumab (λ)	Dupilumab (κ)	Durvalumab (κ)	Eculizumab (κ)
Efalizumab (κ)	Emactuzumab (κ)	Erenumab (λ)	Erlizumab (κ)
Etesevimab (κ)	Fepixnebart (κ)	Gantenerumab (κ)	Gevokizumab (κ)
Golimumab (κ)	Guselkumab (λ)	Ibalizumab (κ)	Ibritumumomab (κ)
Idarucizumab (κ)	Imalumab (κ)	Infliximab (κ)	Ipilimumab (κ)
Isatuximab (κ)	Ixekizumab (κ)	Izalontamab (κ)	Lampalizumab (κ)
Lanadelumab (κ)	Lebrikizumab (κ)	Ligelizumab (κ)	Lumretuzumab (κ)
Matuzumab (κ)	Metelimumab (κ)	Mevonlerbart (κ)	Motavizumab (κ)
Muromonab (κ)	Necitumumab (κ)	Nirsevimab (κ)	Nivolumab (κ)
Obinutuzumab (κ)	Ofatumumab (κ)	Ogalvibart (κ)	Olokizumab (κ)
Omalizumab (κ)	Omburtamab (κ)	Ontamalimab (κ)	Opicinumab (κ)
Orilanolimab (κ)	Panitumumab (κ)	Paridiprubart (κ)	Pateclizumab (κ)
Pembrolizumab (κ)	Pertuzumab (κ)	Ponezumab (κ)	Prezalumab (κ)
Quilizumab (κ)	Radretumab (κ)	Ramucirumab (κ)	Ranibizumab (κ)
Ravagalimab (κ)	Regdanvimab (λ)	Rituximab (κ)	Rontalizumab (κ)
Rozanolixizumab (κ)	Ruplizumab (κ)	Secukinumab (κ)	Serplulimab (κ)
Sifalimumab (κ)	Spesolimab (κ)	Suvratoxumab (κ)	Talacotuzumab (κ)
Tanezumab (κ)	Teneliximab (κ)	Tezepelumab (λ)	Tislelizumab (κ)
Tixagevimab (κ)	Toripalimab (κ)	Tralokinumab (λ)	Trastuzumab (κ)
Tremlimumab (κ)	Urelumab (κ)	Ustekinumab (κ)	Utomilumab (λ)
Vanucizumab (λ)	Vonlerolizumab (κ)	Zenocutuzumab (κ)	

Supplementary Table 2 The 119 clinical-stage therapeutics (CSTs, 103 x κ, 16 x λ) with 100% sequence identity solved crystal structures publicly released on or before 31st July 2021 (ABodyBuilder2's training set cutoff date). Models of these therapeutics were excluded in benchmarking studies. Res: Resolution.

TAP Property	Amber Flag Region	Red Flag Region
L_{tot}	37 (*0*) $\leq L_{tot} \leq$ 42 (*0*)<br>53 (*-2*) $\leq L_{tot} \leq$ 62 (*-1*)	$L_{tot} <$ 37 (*0*)<br>$L_{tot} >$ 62 (*-1*)
PSH	95.58 (*0*) $\leq$ PSH $\leq$ 109.51 (*-0.60*)<br>162.15 (*-5.91*) $\leq$ PSH $\leq$ 195.68 (*-5.91*)	PSH < 95.58 (*0*)<br>PSH > 195.68 (*-5.91*)
PPC	1.28 (*-0.04*) $\leq$ PPC $\leq$ 4.22 (*-0.13*)	PPC > 4.22 (*-0.13*)
PNC	1.94 (*-0.06*) $\leq$ PNC $\leq$ 4.42 (*0*)	PNC > 4.42 (*0*)
SFvCSP	-30.6 (*0*) $\leq$ SFvCSP $\leq$ -6.0 (*0*)	SFvCSP < -30.6 (*0*)

Supplementary Table 3 Flagging regions across the five TAP developability metrics calculated over the 510 CSTs that are modeled with higher CDRH3 confidence (*i.e.* the CST_{conf} dataset). Differences from the CST_{all} guidelines are provided in the brackets. L_{tot}: Total CDR Length; PSH: Patches of Surface Hydrophobicity; PPC: Patches of Positive Charge; PNC: Patches of Negative Charge; SFvCSP: Structural Fv Charge Symmetry Parameter.

Metric	All, Mean Variance/3 Runs	κ, Mean Variance/3 Runs	λ, Mean Variance/3 Runs
PSH	10.533	10.455	11.046
PPC	0.004	0.004	0.000
PNC	0.005	0.005	0.007
SFvCSP	0.572	0.597	0.404

Supplementary Table 4 The mean variance recorded for each structure-based TAP developability metric calculated on three repeat ABodyBuilder2 models of all 664 CSTs (column 2), the subset of 576 κ-CSTs only (column 3), and the subset of 88 λ-CSTs only (column 4).

TAP Property	Amber Flag Region	Red Flag Region
PSH	94.85 (*-0.73*) $\leq$ PSH $\leq$ 110.78 (*+0.67*)<br>168.74 (*+0.68*) $\leq$ PSH $\leq$ 206.93 (*+5.34*)	PSH < 94.85 (*-0.73*)<br>PSH > 206.93 (*+5.34*)
PPC	1.33 (*+0.01*) $\leq$ PPC $\leq$ 4.31 (*+0.09*)	PPC > 4.31 (*+0.09*)
PNC	2.01 (*+0.01*) $\leq$ PNC $\leq$ 4.42 (*0*)	PNC > 4.42 (*0*)
SFvCSP	-30.60 (*0*) $\leq$ SFvCSP $\leq$ -6.00 (*0*)	SFvCSP < -30.60 (*0*)

Supplementary Table 5 The TAP developability guidelines for structure-dependent metrics set by combining three repeat modelling runs for each of the 664 CST Fvs [2]. Differences from the CST_{all} guidelines are provided in the brackets. PSH: Patches of Surface Hydrophobicity; PPC: Patches of Positive Charge; PNC: Patches of Negative Charge; SFvCSP: Structural Fv Charge Symmetry Parameter.

CST	TAP Metric	Scores Across Six TAP Repeats	Simulation-mean Score
Boserolimab	PSH	[124.69,136.15,110.53,118.09,136.09,115.96]	123.04
	PPC	[0.13,0.13,0.14,0.13,0.12,0.13]	0.06
	PNC	[0.30,0.44,0.30,0.30,0.45,0.13]	0.42
	SFvCSP	[-2.00,-1.00,-2.00,-2.00,-1.00,-2.00]	-4.41
Burosumab	PSH	[129.61,128.31,129.63,120.79,127.91,128.47]	145.08
	PPC	[0.02,0.02,0.02,0.02,0.02,0.02]	0.02
	PNC	[0.40,0.39,0.41,0.41,0.40,0.40]	0.28
	SFvCSP	[1.10,1.10,1.10,1.10,1.10,1.10]	2.37
Casdozokitug	PSH	[180.14,182.25,186.93,189.19,188.05,187.97]	202.29
	PPC	[0.05,0.04,0.00,0.04,0.00,0.00]	0.04
	PNC	[0.13,0.19,0.13,0.15,0.13,0.13]	0.18
	SFvCSP	[4.10,4.10,4.10,4.10,4.10,4.10]	6.38
Dostarlimab	PSH	[114.49,114.44,114.46,117.01,117.05,117.07]	128.86
	PPC	[0.00,0.00,0.00,0.00,0.00,0.00]	0.00
	PNC	[0.06,0.06,0.06,0.06,0.06,0.09]	0.05
	SFvCSP	[0.00,0.00,0.00,-2.10,-2.10,-2.10]	-2.63
Glenzocimab	PSH	[158.15,162.54,150.05,131.22,150.29,138.40]	176.08
	PPC	[0.05,0.04,0.05,0.04,0.04,0.04]	0.04
	PNC	[0.00,0.04,0.00,0.00,0.00,0.00]	0.00
	SFvCSP	[3.41,3.10,3.41,6.20,0.00,0.00]	2.47
Golocdacimig	PSH	[160.27,157.61,155.77,153.97,165.92,159.14]	158.89
	PPC	[0.07,0.07,0.07,0.07,0.21,0.07]	0.23
	PNC	[1.64,1.62,1.63,1.59,1.158,1.64]	1.61
	SFvCSP	[-0.80,0.20,-0.80,-0.80,-0.70,-0.80]	-0.19
Simaravibart	PSH	[176.01,163.34,162.64,163.83,179.57,179.66]	168.27
	PPC	[1.50,1.52,1.49,1.52,1.46,1.53]	0.95
	PNC	[0.50,0.51,0.53,0.51,0.49,0.50]	0.20
	SFvCSP	[0.00,0.00,0.00,0.00,0.00,0.00]	1.50
Belimumab	PSH	[154.82,159.33,153.58,160.35,153.37,165.80]	165.52
	PPC	[0.41,0.41,0.40,0.40,0.40,0.38]	0.31
	PNC	[0.17,0.14,0.17,0.17,0.18,0.38]	0.10
	SFvCSP	[-7.98,-7.98,-7.98,-7.98,-7.98,-7.98]	-7.59
Erenumab	PSH	[183.02,183.43,185.25,185.13,183.47,183.25]	185.59
	PPC	[1.49,1.49,1.49,1.49,1.49,1.49]	1.32
	PNC	[0.00,0.00,0.00,0.00,0.00,0.00]	0.00
	SFvCSP	[20.40,20.40,20.40,20.40,20.40,20.40]	12.08
Fepixnebart	PSH	[151.60,151.75,143.12,137.72,153.63,147.53]	174.71
	PPC	[0.04,0.04,0.04,0.04,0.04,0.04]	0.34
	PNC	[0.11,0.11,0.11,0.11,0.12,0.12]	0.11
	SFvCSP	[8.80,8.80,8.80,8.80,8.80,8.80]	6.95
Imalumab	PSH	[140.69,140.68,140.74,140.75,140.69,140.71]	147.27
	PPC	[0.00,0.00,0.00,0.00,0.00,0.00]	0.00
	PNC	[0.07,0.07,0.07,0.07,0.07,0.07]	0.05
	SFvCSP	[0.00,0.00,0.00,0.00,0.00,0.00]	6.13
Quilizumab	PSH	[149.92,146.14,141.97,141.89,141.95,147.65]	148.38
	PPC	[0.04,0.04,0.04,0.04,0.04,0.04]	0.03
	PNC	[0.16,0.16,0.16,0.16,0.16,0.15]	0.31
	SFvCSP	[-10.20,-10.20,-10.20,-10.20,-10.20,-10.20]	-25.22
Regdanvimab	PSH	[185.55,165.15,184.08,180.47,172.50,172.84]	172.31
	PPC	[1.71,1.63,1.63,1.58,1.67,1.67]	1.75
	PNC	[1.53,1.53,1.53,1.53,1.52,1.53]	1.24
	SFvCSP	[0.00,0.00,0.00,0.00,0.00,0.00]	-3.12
Secukinumab	PSH	[153.68,155.74,150.56,152.43,153.79,154.09]	161.34
	PPC	[0.00,0.00,0.00,0.00,0.00,0.00]	0.05
	PNC	[0.13,0.09,0.09,0.13,0.09,0.13]	0.15
	SFvCSP	[0.20,0.20,0.20,0.20,0.20,0.20]	-1.52

Supplementary Table 6 Scores/amber flags (orange text) for repeat ABodyBuilder2 runs and the MD simulations.

Gene Family	# CSTs	% Abundance Amongst these CSTs	% Abundance Amongst Natural Sequences
LV1	24	33.80	29.73
LV2	15	21.13	28.71
LV3	25	35.21	28.11
LV4	0	0	3.08
LV5	0	0	0.94
LV6	3	4.23	3.08
LV7	3	4.23	3.17
LV8	1	1.41	1.82
LV9	0	0	0.55
LV10	0	0	0.80

Supplementary Table 7 Comparison of gene family usages across human gene-derived λ-CSTs, and gene family uses across natural paired sequences from OAS [4].

Gene	# CSTs	% Abundance Amongst these CSTs	% Abundance Amongst LV2 Natural Sequences
LV2-8	0	0	13.83
LV2-11	3	20	11.62
LV2-14	10	66.67	42.85
LV2-18	0	0	13.83
LV2-23	2	13.33	17.87

Supplementary Table 8 Comparison of gene usages across human LV2 gene family-derived λ-CSTs, and gene uses across human LV2-derived natural paired sequences from OAS [4].

CST	PDB Structure Used for Constant Region Grafting (chain IDs, heavy + light)
Belimumab	5Y9K (HL)
Boserolimab	8DS5 (CB)
Burosumab	7VEN (BA)
Casdozokitug	7ZXK (HL)
Dostarlimab	7WSL (HL)
Erenumab	6UMH (HL)
Fepnixnebart	5KN5 (AB)
Glenzocimab	7R58 (HL)
Golocdacimig	7R8U (HL)
Imalumab	6FOE (HL)
Quilizumab	3HR5 (HL)
Regdanvimab	7CM4 (HL)
Secukinumab	6WIO (AB)
Simaravibart	7SBU (HL)

Supplementary Table 9 Protein Data Bank IDs and chain IDs mapping to the coordinates used for CH1/CL domain grafting for each clinical-stage therapeutic (CST).

Stage	Ensemble	Restrained Atoms	Restraint Strength (kJ mol^{-1} nm^{-2})	Duration	Start T (K)	End T (K)
1	Minimisation	Protein Heavy	4184.00	5000 steps	-	-
2	NVT	Protein Heavy	4184.00	0.2 ns	100	300
3	NPT	Protein Heavy	4184.00	0.2ns	300	300
4	NPT	Protein Heavy	2092.00	0.5ns	300	300
5	Minimisation	Backbone Heavy	2092.00	5000 steps	300	300
6	NPT	Backbone Heavy	2092.00	0.2ns	300	300
7	NPT	Backbone Heavy	418.40	0.2ns	300	300
8	NPT	Backbone Heavy	41.84	0.2ns	300	300
9	NPT	-	-	1ns	300	300

Supplementary Table 10 Equilibration protocol steps. Where start and end temeperatures differ, the temperature was linearly increased over the duration of the stage. T: Temperature.

Supplementary References

[1] Lefranc, M.-P., Pommié, C., Ruiz, M., Giudicelli, V., Foulquier, E., Truong, L., Thouvenin-Contet, V., Lefranc, G.: IMGT unique numbering for immunoglobulin and T cell receptor variable domains and Ig superfamily V-like domains. Dev Comp Immunol **27**(1), 55–77 (2003) https://doi.org/10.1016/s0145-305x(02)00039-3

[2] Abanades, B., Wong, W.K., Boyles, F., Georges, G., Bujotzek, A., Deane, C.M.: ImmuneBuilder: Deep-Learning models for predicting the structures of immune proteins. Commun Biol **6**, 575 (2023) https://doi.org/10.1038/s42003-023-04927-7

[3] Leem, J., Dunbar, J., Georges, G., Shi, J., Deane, C.M.: ABodyBuilder: Automated antibody structure prediction with data–driven accuracy estimation. mAbs **8**(7), 1259–1268 (2016) https://doi.org/10.1080/19420862.2016.1205773

[4] Olsen, T.H., Boyles, F., Deane, C.M.: Observed Antibody Space: A diverse database of cleaned, annotated, and translated unpaired and paired antibody sequences. Protein Sci **31**(1), 141–146 (2022) https://doi.org/10.1002/pro.4205
