# Supplementary material for: Contextualising the developability risk of antibodies with lambda light chains using enhanced therapeutic antibody profiling
Source: Commun Biol. 2024 Jan 8;7:62. doi: 10.1038/s42003-023-05744-8 (PMC10774428; doi:10.1038/s42003-023-05744-8)
Supplement: Supplementary file 3 — Description of Additional Supplementary Files [file 42003_2023_5744_MOESM3_ESM.pdf]

Description of Additional Supplementary Files

File name: Supplementary Data 1
Description: Numerical source data for all figures and tables.
